# Assessing the impact of voluntary sustainability standards in Amazonian enterprises involved in the açaí value chain

**DOI:** 10.1016/j.heliyon.2024.e34157

**Published:** 2024-07-06

**Authors:** Kairo Fernandes Martins, Denilson Teixeira

**Affiliations:** a Federal University of Goiás (UFG), Brazil; b National Institute of Metrology, Quality and Technology (INMETRO), Brazil

**Keywords:** Voluntary sustainability standards, Global value chains, Non-timber forest product, Amazon, Açaí

## Abstract

The diversity of sustainable certifications raises questions about the credibility, intentions, and impacts of Voluntary Sustainability Standards (VSS) on Global Value Chains (GVC). Few studies show the impacts of VSS on different sustainable dimensions in sectors such as the non-timber forest product (NTFP) sector. This paper aims to investigate in the value chain of the most important NTFP in the Amazon, açaí, whether VSS contributes to sustainable outcomes in the Governance, Environmental, Economic, and Social dimensions. Using case studies in enterprises of the açaí chain and the use of tools and indicators was possible to generate information that is currently scarce for NTFPs in the Amazon from the VSS perspective. The results show that there is a great distance that the weakest links of the GVC (Micro, Small, and Medium Enterprises – MSMEs) must walk to adopt VSS and be inserted into the global market. The requirements are based on bureaucratic management activities, which are extraordinarily complex and involve many issues and indicators. The VSS lacks supplements that evaluate and validate the results reported by the companies as sustainable. Finally, the VSS is still far from ensuring an inclusive and fully sustainable chain by itself.

## 1 Introduction

Sustainability assessment has often been a field of study and research to help decision-making and environmental, economic, and social policies. As a result, much research has focused on how to significantly measure the sustainability in global value chains - GVCs [[1], [2], [3], [4], [5]].

The linking of GVCs with the sustainable development theme provides the opportunity for the Voluntary Sustainability Standards (VSS) to contribute significantly to sustainable chains. However, the diversity of sustainable standards raises questions about the credibility, intentions, and positive and negative impacts of VSS adoption on value chains and the consumption of certified products and services.

The potential of VSS to make trade more sustainable depends on two crucial components: first, that they generate impact on the base of GVCs, and second, that they are widely used [6]. Numerous surveys have confirmed the positive impacts of VSS in many value chains [[7], [8], [9], [10], [11]]. But other studies have shown that in some cases, the impacts of VSS are not fully proven, or they are more negative than positive [6,[12], [13], [14]]. There are products impacted by VSS that need further study, such as Non-Timber Forest Products (NTFPs), especially if they are Amazonian products [15].

The açaí, a typical fruit from the Amazon, is considered, according to the Amazon Environmental Research Institute – IPAM, the most important NTFP in the region [16]. The fruit has economic, environmental, and social relevance for that region [17]. Açaí based products are examples of exported goods impacted by VSS [15].

In the last decades, the açaí gained significant access to markets outside the Amazon, mainly for its nutritional characteristics, high content of fiber and antioxidants, and for being used as an ingredient in important industries: food and beverages, pharmaceutical, perfumery, and cosmetics [18,19]. For this reason, this value chain was chosen for the case studies in this research from the VSS perspective. The objective is to assess whether the governance, environmental, economic, and social dimensions of the VSS contribute to sustainable outcomes in the açaí value chain.

## 2 Methods

### 2.1 Selection of companies for the study

Initially, 15 companies in the açaí value chain were selected and invited to participate in the research. Of these, 5 companies (of different sizes) collaborated with the study by participating in the interviews and answering the questionnaires.

Besides their different sizes, the companies are from different segments of the chain (extracting cooperatives, processing companies, distributors). All are in the Amazon region. Among the companies, two were included because they are in the process of adopting some VSS or have shared certification through partnerships with large companies, the microenterprise, and the small cooperative, respectively. The inclusion criterion was to have at least one VSS or are in the process of adopting one.

The small traders, popularly known as açaí beaters, who process the fruit for consumption on the day (or freeze it for travel) and sell it at fixed points easily found in the cities of the Amazon region, were excluded. It was decided to exclude them because this group of traders does not have or did not want to provide information about the origin of the fruit, not being possible to identify if the fruit comes from producers or suppliers certified by any VSS.

Because this is sensitive, strategic information that involves market competition, the names of the companies have been mischaracterized to preserve the image and the data. Table 1 shows the code and information on participating enterprises in the study.

**Table 1:** Information on participating enterprises.

Company size	Company code	Opening year	Position in the açaí GVC
Micro enterprise	E1MI	2011	Extractive and processor
Small Cooperative	E2CP	2015	Extractive
Medium Co-op	E3CM	2017	Extractive and processor
Tranding Company	E4ME	2009	Exporter
Multinational	E5GR	2005	Processor and exporter

### 2.2 Selection of methodological tools

The increased importance of sustainability assessment in value chains can be seen in several studies [12,14,[19], [20],]. For [21], the notion of sustainability has become a normative guiding principle for evaluating systems (food systems, for example). For this reason, several holistic sustainability assessment methods have been developed, including the Food and Agriculture Organization (FAO) of the United Nations, which has published guidelines for these types of assessments, called the Sustainability Assessment of Food and Agriculture Systems or SAFA Guidelines. SAFATool was the tool used in the study, adapting the SAFA guidelines into worksheets containing the questionnaires and indicators for collecting data from the companies [22].

The SAFA Guidelines provide a hierarchical framework of four dimensions (good governance, environmental integrity, economic resilience, and social welfare). Within each dimension, there are themes, sub-themes, and indicators. An absolute objective describes the target state of sustainability for each sub-theme [22]. In their work [2,19], considered that the SAFATool can be conceptualized as Multi-Criteria Analysis (MCA) for each sub-theme of the SAFA Guidelines. The relationship of the four dimensions provides a visible and clear result by the so-called sustainability polygon.

The choice of the SAFATool tool was due to the dimensions worked on and the themes associated with the Voluntary Sustainability Standards. The four dimensions of the SAFA tool contain 21 themes, 58 sub-themes, and 116 indicators. All these contents were adapted into 4 electronic worksheets distributed to the companies and used to conduct the meetings and interviews. The overview of SAFA indicators by sub-themes, themes, and dimensions can be seen in Appendix. The adapted questionnaires for each dimension can be found in the supplementary materials. The animal welfare theme was excluded from the study because its sub-themes and its five indicators are not related to the sector. As a result, 20 themes and 111 indicators were used in the data collection and analysis.

### 2.3 Data analysis techniques

After the interviews, the data were entered into the SAFA tool. Then it was performed the Multi-Value Qualitative Comparative Analysis – MVQCA [[23], [24], [25]]. Finally, SWOT Analysis [26] was used to synthesize the impacts of VSS on the açaí value chain.

Due to the Covid-19 pandemic, interviews were carried out remotely using Google Meet, Microsoft Teams and Zoom tools. In the interviews, the representatives answered YES or NO to the questions based on the VSS contribution to each indicator. The “Quantification/Observations” field was for free responses, in which, when possible, respondents quantified the answer in percentages, units or added other information they thought relevant about the indicator.

In the “Quality of data/information” field, the company classified the information as high, medium, or low quality. In the “Evaluation” field, the companies assigned a score on a scale of 0–5. Table 2 shows the data quality score and the concept for each rating.

**Table 2:**
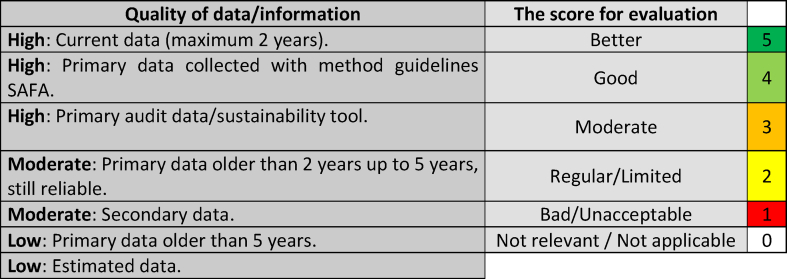
Quality of information and score for evaluation.

## 3 Results

### 3.1 VSS most adopted by companies in the açaí value chain

The five companies in the study have between two and fourteen VSS. A total of 28 different certifications were mapped for the açaí chain by analyzing the 15 companies invited. Of the 28 VSS, 11 (39 %) are to supply the organic market in different countries, and 7 (25 %) are related to food safety. Almost half of the consulted companies in the certified açaí chain (48 %) adopt VSS for organic. 19.5 % of the companies also adopt VSS for food safety.

Table 3 shows, in chronological order of appearance, the main information of each VSS adopted in the açaí GVC: name, year of appearance, product/sector categories to which the VSS apply, main nations where they are found, and the Organization that operates them. The number in parentheses in the first column represents the number of enterprises that adopt VSS among the surveyed companies.

**Table 3:** Voluntary Sustainability Standards adopted in the Global Açai Value Chain.

VSS name	Sustainable certification/Ecolabel	Year	Product/Sector categories this standard applies to	Main countries where this VSS is found	Managing organization/Type
Australian Certified Organic (1)		2002	Cosmetics/Personal care, Fish/Fisheries and Food.	Cook Islands	Australian Certified Organic - subsidiary of Biological Farmers of Australia, Non-profit
B Corporation (1)		2007	Appliances, Building products, Carbon offsets, Cleaning products, Cosmetics/Personal care, Electronics, Energy, Financial services, Fish/Fisheries, Food, Forest products/Paper, Health care services & equipment, Machinery & Equipment, Packaging, Professional, scientific and technical services, Textiles, Tourism, Transportation, Waste management & Recycling, Water; Other.	Canada, United States	B Lab, Non-profit
Bio Suisse (1)		1981	Aquaculture, Bananas, Cereals, Cocoa, Coconut (Fresh), Coffee, Cotton & fibers, Fisheries, Flowers, Food & Beverages, Fruits, Health, Honey, Households, Jewelry, Nuts, Other products, Palm oil, Plants, Rice, Soy, Spices, Sugar, Tea, Vegetables, Wild fishes.	Liechtenstein, Switzerland	Bio Suisse, Non-profit
Brand Reputation Compliance – BRCGS (2)		1996	Aquaculture, Bananas, Cereals, Cocoa, Coconut (Fresh), Coffee, Cotton & fibers, Fisheries, Floriculture products, Flowers, Food & Beverages, Fruits, Honey, Households, Jewelry, Nuts, Other products, Palm oil, Plants, Rice, Soy, Spices, Sugar, Tea, Vegetables, Wild fishes.	Different countries on five continents.	Private entity (e.g. an NGO, an industry, an association, or a company).
Canada Organic (4)		2009	Food	Canada	Canadian Food Inspection Agency - Government
Fair For Life (6)		2006	Aquaculture, Clothing, Cosmetics, Detergents, Food & Beverages, Fruits, Health, Households, Housing, Other products, Palm oil, Textiles/Garment, Toys, Wild fishes and Tourism.	Present in several countries in Africa, America, Asia and Europe.	IMO Group - For-profit
Forest Stewardship Council (FSC) - Chain of Custody Certification (1)		1993	Health, Households, Housing, Other products, Plants	Different countries on five continents.	The Forest Stewardship Council® (FSC), Non-profit
Forest Stewardship Council® (FSC) - Forest Management Certification (1)		1994	Forest products/Paper	Different countries on five continents.	The Forest Stewardship Council® (FSC), Non-profit
Forest Stewardship Council® (FSC) - Ecosystem Services Procedure (1)		2018	Forestry	Different countries on five continents.	The Forest Stewardship Council® (FSC), Non-profit
Food Safety System Certification - FSSC 22000 (4)		2009	Aquaculture, Catering, Cosmetics, Food & Beverages, Fruits, Households, Jewelry, Other products, Wild fishes.	Different countries on five continents.	Private entity (e.g. an NGO, an industry, an association, or a company).
Halal (5)		2004	Food, clothing, tourism, media/leisure, pharmaceuticals and cosmetics.	Indonesia, Turkey and Pakistan are the biggest markets, South and Southeast Asia, European Union countries, mainly France, Germany and England, Canada, Brazil (mainly for meats).	FAMBRAS HALAL Certification LTD. - For-profit
Hazard Analysis and Critical Control Point – HACCP (4)		The 90's	Food (primary production to food distribution). The certification is also directed towards food-oriented packaging, regardless of the size of the company, and promotes food safety.	world recognized	not applicable
IBD NON GMO (1)		–	Agriculture, Livestock, Processing and Cosmetics	Brazil and more than 20 countries.	QIMA/IBD
IBD Organic (2)		1995	Agriculture, livestock, fibers, aquaculture, processing, inputs, extractivism, cosmetics, wines and cleaning products.	Brazil and more than 20 countries.	QIMA/IBD
IFS Food - International Featured Standards (2)		2003	Aquaculture, Bananas, Cereals, Clothing, Coconut (Fresh), Fisheries, Flowers, Food & Beverages, Fruits, Honey, Households, Jewelry, Nuts, Other products, Palm oil, Plants, Rice, Soy, Spices, Sugar, Tea, Vegetables, Wild fishes.	Present in several countries in Africa, America, Asia and Europe.	Private entity (e.g. an NGO, an industry, an association, or a company).
ISO 22000 (2)		2005	Food	world recognized	International Organization for Standardization - ISO
Japanese Agricultural Organic Standard (JAS) (3)		2000	Food	Japan	Japanese Ministry of Agriculture, Forestry and Fisheries - MAFF
Korea Organic Programs/MAFRA Korea Organic (1)		2013	Food	Korea	Ministry of Agriculture, Food and Rural Affairs - MAFRA
Kosher Parve (7)		The 80's	Food (espresso coffee, tea, confectionery, bakery, yogurt, chocolate, chocolate, biscuits and cookies, sweets, snacks, cereal bars, etc.), honey, drinks (energy, alcoholic and non-alcoholic), condiments, fruits (frozen), dehydrated, in syrup, pulp), frozen vegetables, sugars and sweeteners, various preserves, flours, bran, grains and oils.	Israel, United States, Argentina and European countries.	Kosher, granted by a rabbinic agency
NON-GMO Project Verified (2)		2007	Food	Canada, United States.	NON-GMO Project, Non-profit
Organic Brazil (11)		2003	Food	Brazil	Ministry of Agriculture, Livestock and Supply - MAPA
Organic Farming -EU/EU Organic Products Label (7)		1991	Aquaculture, Bananas, Cereals, Cocoa, Coconut (Fresh), Coffee, Cosmetics, Cotton & Fibers, Flowers, Food & Beverage, Fruits, Health, Honey, Nuts, Palm Oil, Plants, Rice, Soybeans, Spices, Sugar, Tea, Vegetables, Fish.	Different countries on five continents.	European Commission
Organis (1)		2005	Food, chemical products, health inputs, beverages, fertilizers, sweeteners, consulting services, certifiers, cosmetics, flours and others.	Brazil	Organis, Non-profit
Orgánico México (1)		2014	Food	Mexico	Servicio Nacional de Sanidad, Inocuidad y Calidad Agroalimentaria - SENASICA
UN Global Compact (2)		2000	Administration, Aquaculture, Bananas, Biofuel, Carbon offsets, Catering, Cereals, Clothing, Cocoa, Coconut (Fresh), Coffee, Cosmetics, Cotton & fibers, Eco-system services, Electricity or gaz, Electronics, Finance, Fisheries, Flowers, Food & Beverages, Fruits, Health, Honey, Households, Housing, Jewelry, Natural input, Nuts, Other products, Other service activities, Palm oil, Plants, Rice, Science, Social work, Soy, Spices, Sugar, Tea, Textiles/Garment, Tourism, Toys, Transport, Vegetables, Water supply, Wholesale and retail trade, Wild fishes.	Different countries on five continents.	Public entity (e.g. governmental agency).
Vegan Product - SVB (2)		2013	Food, Cosmetics, Textiles, Cleaning products.	Brazil	Brazilian Vegetarian Society - SVB
Amapá Seal - Product of the Middle of the World (2)		2018	Miscellaneous foods, beverages, construction, animal feed, herbal medicines.	Brazil	Amapá Economic Development Agency
USDA National Organic Program – NOP (10)		1990	Bananas, Cereals, Cocoa, Coconut (Fresh), Coffee, Cotton & fibers, Flowers, Fruits, Honey, Nuts, Other products, Palm oil, Plants, Rice, Soy, Spices, Sugar, Tea, Vegetables.	United States	United States Department of Agriculture - USDA

### 3.2 Case study 1: company E1MI - microenterprise

E1MI is a family microenterprise that, in addition to producing açaí, works in the agro-industry and commerce of andiroba, copaiba, vegetable oils, manioc flour, and other typical Amazonian fruits. The company currently does not have VSS and is getting ready to adopt the sustainable certification *Orgânico Brasil*, Fair Trade, and USDA (Organic for the United States) and has started the analysis of the adhesion term for the Halal certification (VSS that follows the Islamic principles).

**Fig. 1:**
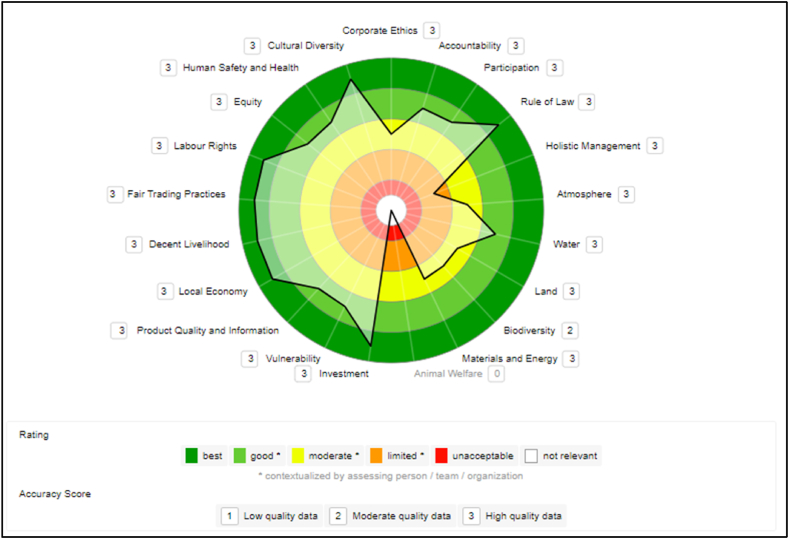
E1MI microenterprise sustainability polygon.

The data collected at the E1MI company is mostly of high quality (primary and current data), except for one indicator of the “Biodiversity” theme in the Environmental dimension, considered of moderate quality (secondary data), according to the SAFA Tool classification. The sustainability polygon (Fig. 1), generated with the collected data, displays the results per theme in the sustainable dimensions. These results reflect the improvements perceived by the microenterprise with the preparations and changes introduced to adapt to the VSS that it intends to adopt.

In the Governance dimension, based on the improvements made and ongoing projects, the company had, according to the SAFATool criteria, a high performance (maximum score of “best”) in the “Rule of Law” theme and good performance in the “Accountability” and “Participation” themes. The “Corporate Ethics” theme was rated as “moderate”. In contrast, the “Holistic Management” theme was the weakest point in this dimension and was rated as “limited."

For E1MI, the best-rated theme in the Environmental dimension was “Water”, which was rated “Good”. The company obtained moderate ratings in the other themes “Atmosphere”, “Earth”, “Biodiversity”, and “Materials and Energy”. However, there are several indicators for which the company does not have data. Since E1MI is a microenterprise, the SAFA tool makes weightings on micro and small producers to consider (in the evaluation) the different levels of influence of the companies. Therefore, when a company is declared or classified with these sizes in SAFATool, the calculation of the scores includes exemptions for the indicators. This is only valid within the Environmental dimension.

If one of these indicators is not answered by the small producer, the related sub-theme will not rate the omissions with an “unacceptable” score and will be classified as “No data”. The answer is evaluated as neutral in the calculation of the final result. According to FAO [22], larger companies have a potentially greater sphere of influence than small producers. For this reason, SAFATool recognizes the increasing responsibility for sustainable production as the size of the company grows.

E1MI is an expanding company that has made investments that have provided an excellent performance on the Economic dimension for this microenterprise. The themes “Investment” and “Local Economy” scored highest (“best”). For the themes “Vulnerability” and “Product Quality and Information”, it got a “good” rating.

In the Social dimension, E1MI has actions that provide a high performance. It was the dimension where the micro-enterprise performed the best. The themes “Decent livelihoods” and “Fair trade practices”, “Labor rights”, and “Cultural diversity” achieved the highest rating, “best/very good”. The other themes, “Net equity/Equity”, “Security and human health”, got a “good” rating.

Even as a microenterprise, many indicators in this dimension are impressive due to E1MI's attitude and actions with employees, extractive suppliers, and the community. The administrator pointed out that the input suppliers are locals, and the jobs are held by residents of nearby communities. In this way, the company keeps the local economy active, generating jobs, income, and sustainable business in the Amazon.

### 3.3 Case study 2: company E2CP – small cooperative

E2CP is a small cooperative that involves more than 50 families in açaí production. The Cooperative was created by the involvement of the river people of the region, who organized themselves to try to be part of the whole agricultural chain, maintaining the income within the community; currently, the cooperative only acts in the agricultural chain process, planting, and harvesting. The most important product is the açaí “in natura".

**Fig. 2:**
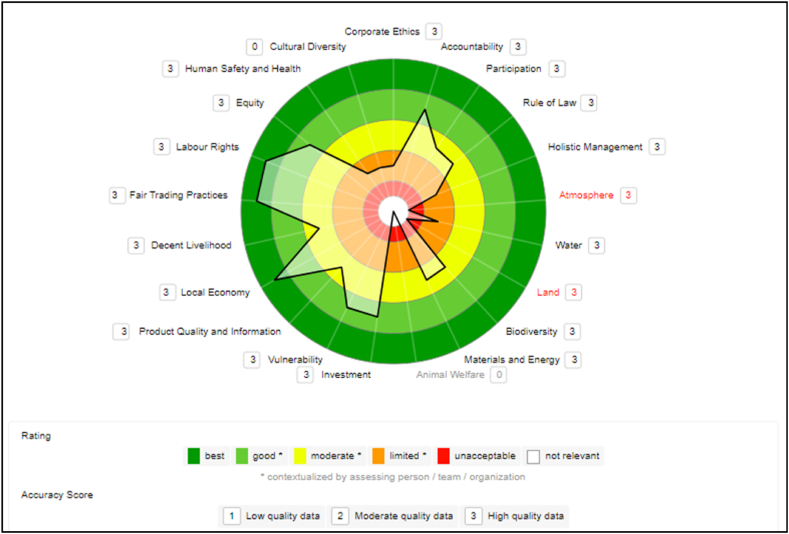
E2CP cooperative sustainability polygon.

E2CP has no VSS in its name. VSS Fair for Life and *Orgânico Brasil* is in the “legal umbrella” (term mentioned by the president of the cooperative) of two large companies in the açaí value chain, and this partnership exists. In addition to these VSS, E2CP is in the early stages of adopting FSC certification, with the first activities beginning in 2023 with a Certification Agency. The data collected in the company E2CP is classified as high quality because all data is primary and current. Fig. 2 shows the generated sustainability polygon.

In the Governance dimension, the theme that stands out in E2CP is “Accountability,” with performance scored as “good.” The company's result is mainly due to the commitment to regularly evaluate and fulfill the mission with the involvement of the interested parties.

The themes “Rule of Law” and “Participation” were considered moderate. As for the themes “Corporate Ethics” and “Holistic Management”, they got the lowest results in this dimension, classified as “limited”. The reason for these medium or lower scores is due to the sub-themes not being well established in the Cooperative, only partially identified and understood.

Even in partnership with large companies for VSS adoption, E2CP's results show weaknesses in the Environmental dimension. For the themes “Atmosphere” and “Earth”, it had an “unacceptable” rating. In the theme “Water”, got a “limited” rating. The best scores were in “Biodiversity” and “Materials and Energy”, still with “moderate” scores.

The results showed that one of the weaknesses is the lack of planning and documented procedures that facilitate the development of plans with targets for their implementation (pollution prevention, water use, soil use, conservation, material and energy use, and waste reduction). There are many plans, only in the realm of ideas, which need to be formalized and implemented procedures.

Sub-themes with performance indicators classified as “No Data” include: “Greenhouse Gases”; “Air Quality”; “Ground and Surface Water Removal”; “Water Pollutant Concentration”; “Wastewater Quality”; “Physical Soil Structure”; “Chemical Soil Quality”; “Materials and Energy” sub-theme indicators, among others.

In the Economic dimension, E2CP scored well, with the theme “Local Economy” achieving the highest score, “best”. For the themes “Investment” and “Vulnerability” the score was “good”. The theme “Product Quality and Information” had the lowest score in the economic dimension, “moderate”, due to half of the indicators scoring between “unacceptable” and “limited”. The company's investments were focused internally to improve management.

According to the CEO, to distribute the açaí production, the cooperative members depend exclusively on third-party freight. This increases the price of the product and considerably reduces the families' profits since it is the stage in which the producer starts to decrease his earnings. The *peconheiros* (agro-extractivist), in general, sell their products to an intermediary, who passes it on to a boatman, who sells it to the factories. Since the price of the plant is fixed by the company, it is the extraction worker who loses. Consequently, logistical investments and partnerships to eliminate intermediaries are essential to the Cooperative.

Depending on the theme, there are variations in the evaluation of the E2CP in the Social dimension. The themes “Fair trade practices” and “Labor rights” received the highest rating, “best”. The theme “Equity” was rated as “good” and the theme “Decent livelihood”, “moderate”. However, the themes “Human health and safety” and “Cultural diversity” were rated “limited”. Such variations are due to the company's deficiencies in not having yet deployed and implemented basic actions such as those included in the indicators evaluated.

### 3.4 Case study 3: company E3CM – medium cooperative

**Fig. 3:**
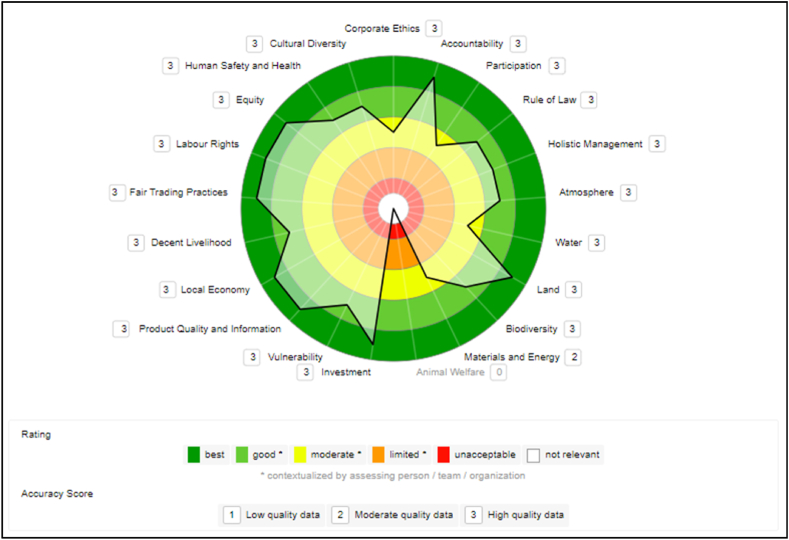
E3CM cooperative sustainability polygon.

E3CM is a medium-sized cooperative. Regarding the açaí chain, the E3CM occupies practically all stages of the chain (handling, collection, processing, transportation, trade, and sale). E3CM currently has eight VSS: Vegan Certified, FSC Handling, FSC Ecosystem Services, FSC Chain of Custody, Amapá Seal, Organic Brazil, Organic for the European Union, and Organic for the United States. For the Cooperative's representatives, the adoption of VSS enabled participation in several markets since the good practices meet the requirements of important economic players (United States, Canada, European countries, etc.). Fig. 3 shows the results in the sustainability polygon.

The data collected in the company E3CM is predominantly of high quality (current primary data or primary audit data), except for one indicator of the theme “Materials and Energy” in the Environmental dimension, which is considered on average to be of low quality (estimated data).

The Governance dimension is very well understood and followed in E3CM. The Cooperative scored highest, “best,” on the theme of “Responsibility” and “good” on “Rule of Law” and “Holistic Management.” Moderate score on “Corporate Ethics” and “Participation".

A good performance in Governance is related to organization, partnership, and process mapping and documentation. Scores were lower in “Corporate Ethics” as improvement is still needed in the sub-theme “Due Diligence”, which is currently in construction of the impact assessment policy to inform decisions that will have long-term impacts in the field of sustainability. In “Participation, the sub-theme “Complaint Procedure” is understood but not well established, lowering the average score for the theme.

In the Environmental dimension, the Cooperative has already noticed improvements with the adoption of VSS. The certifications require improvements and the achievement of goals in the Environmental dimension in several sub-themes and indicators analyzed in the SAFA tool: “Air quality” with “Air pollution reduction target”; “Water quality” with “Water conservation target”; “Ecosystem diversity” with “Landscape preservation plan” and “Genetic diversity” with “Genetic diversity enhancement practices”. With this, E3CM had to adapt and seek improvements besides following the good practice manuals when it chose to adopt the VSS.

In the E3CM, the best-rated theme in the Environmental dimension was “Earth”, achieving the highest score (“best”). The company scored “good” on the “Atmosphere” and “Biodiversity” themes and “moderate” on the other themes, “Water” and “Materials and Energy".

The E3CM had an excellent performance on the themes related to the Economic dimension. It got top scores in “Investment”, “Product Quality and Information”, and “Local Economy”. Besides a “good” rating on “Vulnerability".

The Cooperative also started to commercialize directly with the final consumer using a sales warehouse, aiming to improve the income of the families that survive from açai.

In the Social extractivist dimension, the E3CM had great results. The themes “Fair trade practices”, “Labor rights” and “Equity” received the highest rating, “best”. The other themes, “Decent livelihoods”, “Safety and human health”, and “Cultural diversity” achieved a “good” rating.

According to the managers, among the main indicators evaluated by the SAFA tool in this dimension and very required by VSS for the company to advance and improve are: “Capacity development”, “Health and safety training”, “Public health” and “Food sovereignty”. In addition to the requirements of the VSS, the company follows the Statute and current legislation related to these indicators.

### 3.5 Case study 4: company E4ME - *trading company*

E4ME is a medium-sized Trading Company those exports products based on açaí and other Amazonian fruits. The company has already exported to more than 60 countries. E4MEpossui currently has eight VSS: Company B/Certified B; Organic Farming (EU); Global Pact, and USDA.

**Fig. 4:**
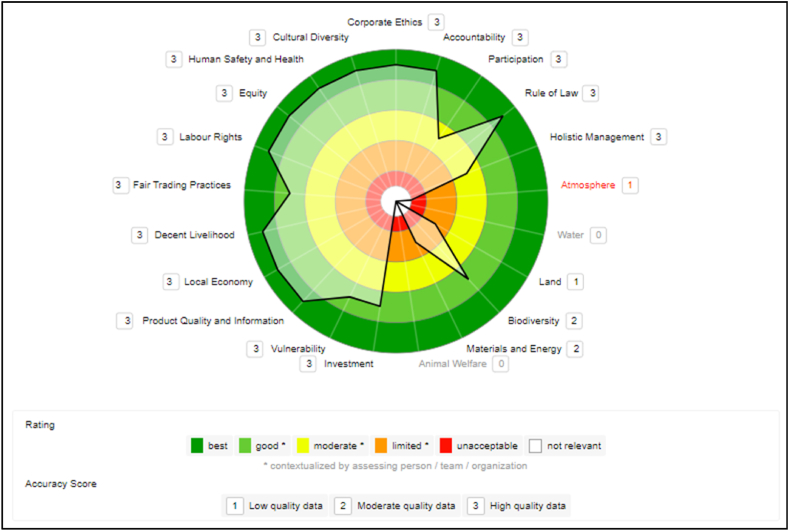
Trading Company E4ME sustainability polygon.

The data collected in the E4ME company is mostly of high quality (current primary data or primary audit data), except for some themes, sub-themes, and indicators from the Environmental dimension. These are not applicable when analyzing the company without the production area, as there was only a commercial office. These themes, sub-themes, and indicators of the Environmental dimension were estimated for the agribusiness inaugurated in 2024, and for this reason, this data is considered of low quality by the methodology (estimated data). Fig. 4 shows the sustainability polygon generated with the data per theme for the Trading Company.

As an export trade intermediary, E4ME, in the Governance dimension, scored highest in the themes “Corporate Ethics”; “Accountability”, and “Rule of Law”. And “moderate” performance on “Participation” and “Holistic Management".

For the analysis of environmental issues, analyzing only E4ME's current activities (as a trading company), it was necessary to exclude the theme “Water”, since there is no production process, only little water consumption in the office. Even so, there are actions to avoid waste on the company's premises.

Consequently, the results for the Trading Company were: “good” rating for “Biodiversity”; “limited” for the themes “Earth” and “Materials and Energy” and “unacceptable” rating for the theme “Atmosphere”, mainly because of the lack of an action plan to address the theme, planned during the inauguration of the agro-industry.

According to the estimated data provided by the company, the results for agribusiness are high performance in the theme “Biodiversity”, with the highest score, “best”; result classified as “good” for the theme “Earth”; moderate results in the themes “Water” and “Materials and Energy” and again “unacceptable” in the theme “Atmosphere”, because the creation of plans in the sub-themes and indicators of this theme is missing”. There is also the goal of the plant being self-sustainable in energy consumption and generation.

In the economic dimension, the highest scores were achieved in the themes “Product Quality and Information” and “Local Economy”. The themes “Investment” and “Vulnerability” scored “good".

The Trading CompanyE4ME had excellent results in the Social dimension. Almost all themes are rated as “best”. The only theme that scored differently was “Fair trade practices”, with a “good” rating.

### 3.6 Case study 5: company E5GR - multinational

E5GR is a multinational company that has two açaí processing plants. In an interview, the administrative manager informed that the company does not own açaí plantation farms, as it acquires the fruit from organizations and cooperatives. The enterprise works to ensure that all the açaí used by the brand is harvested completely “wild,” using low-impact techniques, such as manual harvesting and no pesticides.

E5GR joined 14 VSS: BRCGS *Food Safety*, *Fair for Life*, *Hazard Analysis and Critical Control Point* (HAACP), Halal, *Kosher Parve*, Global Pact, and Amapá Seal. Besides these, the company saw the need for the recognition of açaí as a product of organic origin and also has the VSS: *Australian Certified Organic*, *Canada Organic*, *Japanese Agricultural Organic Standard* (JAS), Organic Brazil, Organic Mexico, Organic for European Union, and USDA.

**Fig. 5:**
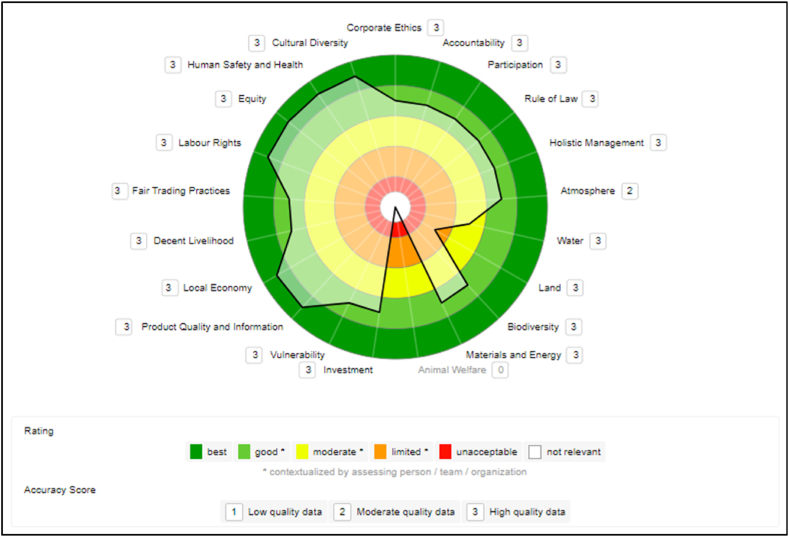
E5GR multinational sustainability polygon.

The data collected in the E5GR company is predominantly of high quality, except for some sub-themes and indicators of the Environmental dimension, which have been classified as of medium quality (primary data from more than 2 years ago). Fig. 5 shows the results in the sustainability polygon.

In the Governance dimension, the five themes (“Corporate Ethics,” “Accountability,” “Participation,” “Rule of Law,” and “Holistic Management”) were all rated well, ranking entirely in the region of the sustainability polygon considered “good."

In E5GR, the themes in this dimension had the following ratings: top ratings in “Atmosphere”, “Biodiversity”, and “Materials and Energy”. On the “Water” theme, the score was “moderate”, and the “Earth” theme had the lowest score on this dimension, “limited".

The fact that E5GR does not have the plantation and fruit extraction stage among its processes, as it acquires açaí from several suppliers, may explain the lower results in the “Earth” theme, in which it was not possible to evaluate which sustainable practices the company provides at the origin. But because it is part of a global value chain and invests in suppliers and extractive communities, it also needs to provide improvements in these other parts of the chain, which supply its main input and track it.

E5GR data in the Economic dimension scored highest (“best” rating) in the themes “Product Quality and Information” and “Local Economy”. The themes “Investment” and “Vulnerability” achieved a “good” rating on the score.

E5GR had excellent results in the Social dimension. The themes “Decent livelihoods” and “Fair trade practices” achieved the rating “good”. The other themes, “Labor rights”, “Equity”, “Safety and human health” and “Cultural diversity” achieved a “best” rating.

### 3.7 Comparative results

The results below show comparisons of the enterprises of the açaí GVC using the sustainability polygons. The overlapping polygons show in which evaluated themes these institutions differ or resemble each other.

#### 3.7.1 Medium Cooperative (E3CM), trading (E4ME) and multinational (E5GR) companies

**Fig. 6:**
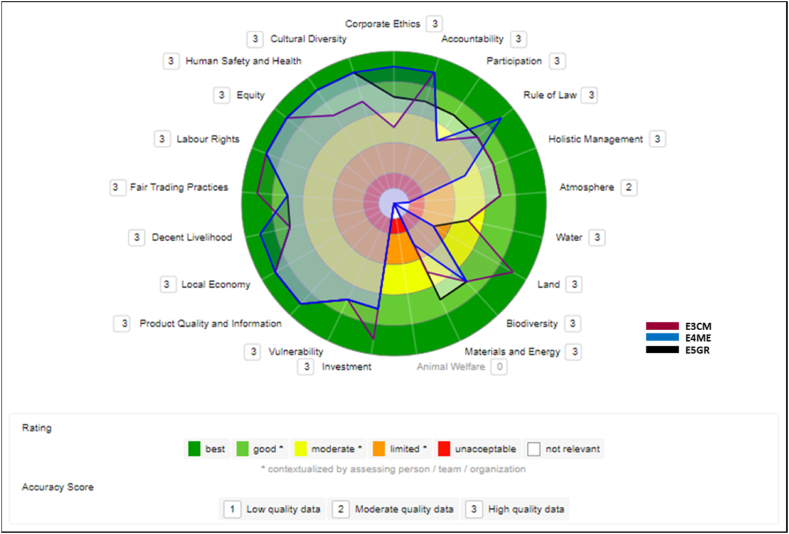
Comparison between E3CM, E4ME, and E5GR companies.

Fig. 6 shows the comparison between the largest companies in size in this study (Medium Cooperative, Trading Company, and Multinational). These firms are generally scoring relatively close and rated well on the economic, social, and governance dimensions. Different from the environmental dimension, where the results, although diverse, had more points lower than the other dimensions.

#### 3.7.2 Micro (E1MI), Medium Cooperative (E3CM) and multinational (E5GR) companies

**Fig. 7:**
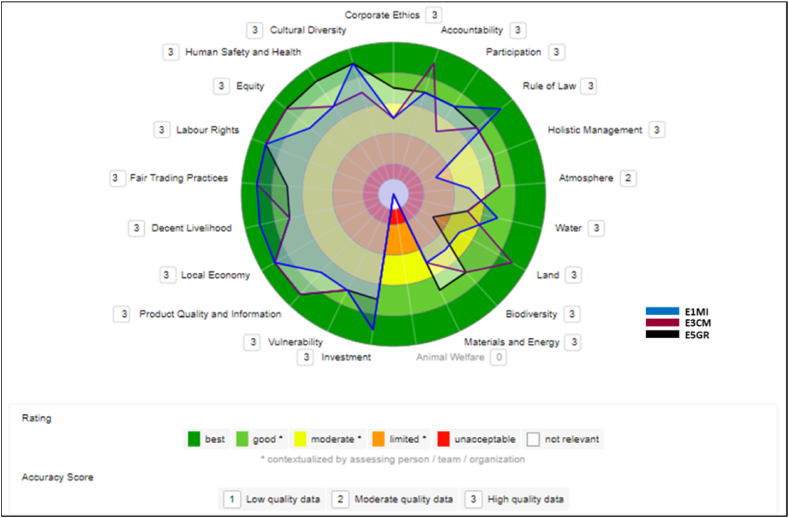
Comparison between E1MI, E3CM, and E5GR companies.

In Fig. 7, the micro-enterprise was compared with the two largest companies in terms of size and amount of VSS adopted, Medium Cooperative and Multinational. The simulation showed that even without VSS, the micro-enterprise performed better or similar to certified companies on many themes.

#### 3.7.3 Micro (E1MI), trading (E4ME) and multinational (E5GR) companies

**Fig. 8:**
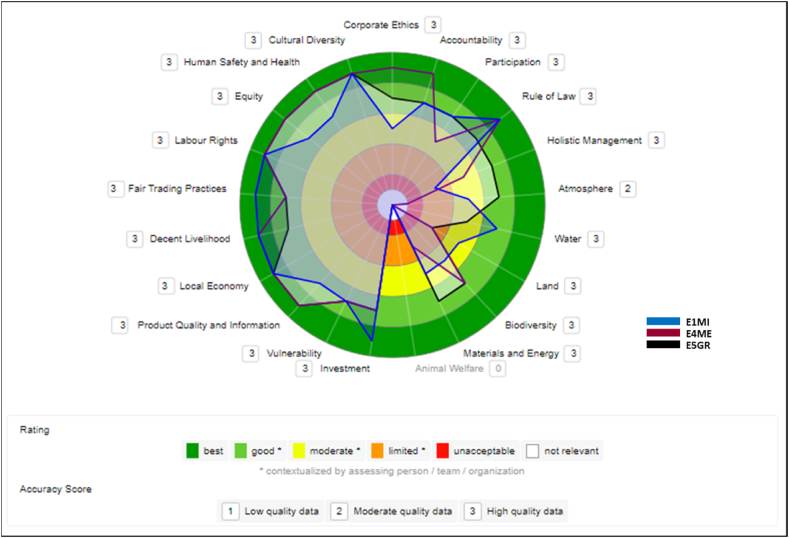
Comparison between E1MI, E4ME, and E5GR companies.

In Fig. 8, we replaced the medium-sized Cooperative with the Trading Company, also medium-sized. The comparative results are not more different from the simulation in Fig. 7. The micro-enterprise continues to score better or equal to certified companies on many themes. The multinational performed very well and maintained a certain constancy of scores across the themes. Within the expected of a large company with many resources, better structuring, and record holder in certifications at GVC.

#### 3.7.4 Small (E2CP) and medium (E3CM) cooperatives

**Fig. 9:**
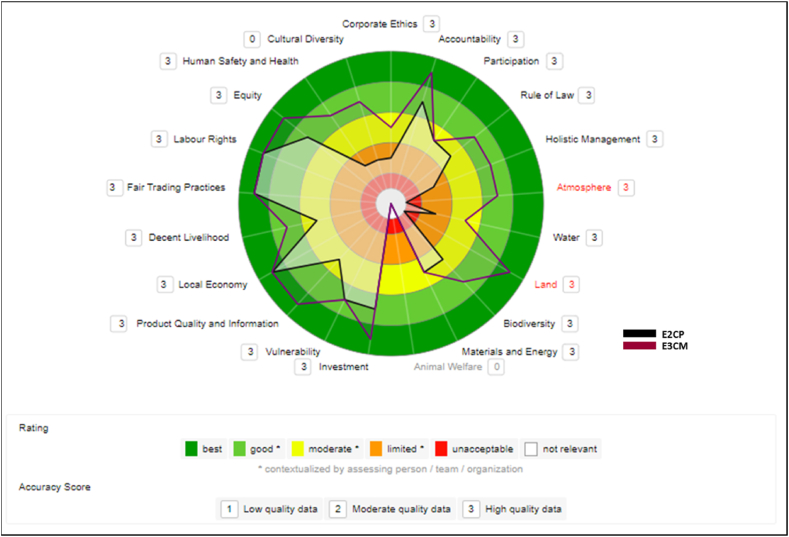
Comparison between E2CP and E3CM cooperatives.

When comparing the two Cooperatives in the study of different sizes (Fig. 9), the results are quite heterogeneous. The small-sized Cooperative had a lower and more distant performance from the medium-sized one. It is important to highlight that not only the structure of the cooperatives is different, a fact that already influences their performance, but also the number of investments and partnerships that each one has, as well as the time they have been dealing with the implementation of sustainable actions. The medium-sized Cooperative has VSS and introduced these sustainable requirements more years ago than the small Cooperative, which started partnerships to adopt VSS recently.

#### 3.7.5 Micro companies (E1MI) and small cooperative (E2CP)

**Fig. 10:**
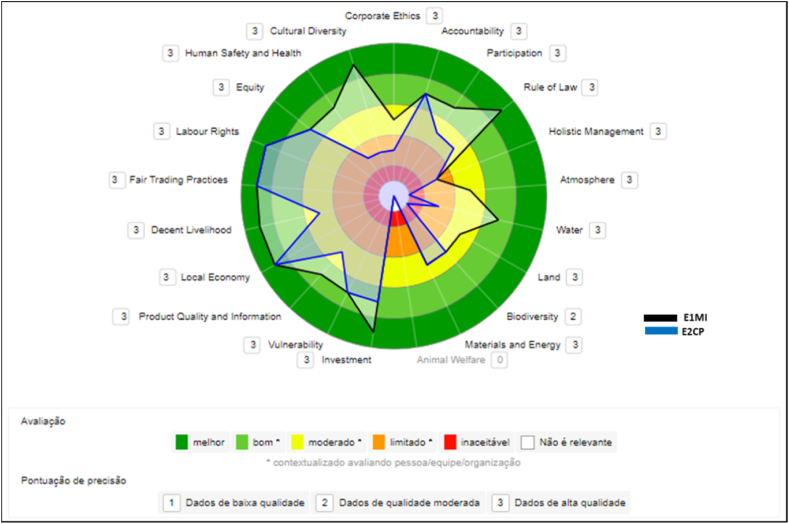
Comparison between E1MI and E2CP companies.

Fig. 10 compares the two smallest companies in the study, which are beginners in the process of adhering to sustainable certifications. Even if the small Cooperative is under the legal umbrella of large firms about VSS, its performance was inferior to that of the micro-company.

After the individual and comparative analyses, the sustainable improvements perceived by the companies related to the VSS, as well as the influences of these certifications on the GVC of açaí, were synthesized in Table 4. To show the impacts of VSS on the açaí GVC comprehensively, considering all the data collected, the SWOT Analysis was performed.

**Table 4:** Summary of sustainable improvements related to VSS perceived by companies and their contributions to the açaí chain.

FIRMS	SPECIFIC RESULTS IN EACH DIMENSION			
	GOVERNANCE	ENVIRONMENTAL	ECONOMIC	SOCIAL
**Micro enterprise (E1MI)**	Partnerships for investment in management.	Ecologically correct extraction.	Investment in improving processes (Good Manufacturing Practices).	Valuing local labor with wages above the minimum wage.
**Small Cooperative (E2CP)**	Organization of production and partnership with large companies to obtain VSS.	Sustainable management practices.	Investment in the organization of cooperative members.	Collective work and fair price with transparent contracts.
**Medium Co-op (E3CM)**	Community Protocol and integrated audits.	Improvements with good practices in the management of açaí with minimal impact.	Investments and donations received from companies (national and international) and direct access to the consumer market at a fair price.	Development of cooperative members, their families and the community.
**Tranding Company (E4ME)**	Mapped and documented processes, responsibility matrix and diagnostic analysis. Purchase and relationship policy.	For suppliers, there is support for management and recovery of areas.	Relationship with suppliers and customers. Traceability (system implementation).	Development of extractive suppliers. Cost allowance for courses and college of employees. Welcoming Indigenous Peoples.
**Multinational (E5GR)**	Sustainability management plan, Impact Report, fair trade.	Actions that resulted in the storage of 12 million metric tons of carbon dioxide per year. Reduction of plastic in packaging.	Investment in VSS for açaí producing cooperatives in exchange for commercial contracts. Base price of açaí defined each year, regardless of market fluctuations.	Rural and urban projects with better infrastructure for small producers, increasing supply partnerships. Study of Biodiversity in communities.
**Results for GVC**	– Efficiency and effectiveness in operational processes.	– Sustainable strategies that raise awareness of environmental preservation.	– Accompaniment of purchase and sale of certified products throughout the chain.	– Transparency in supplier X processor X final consumer relationships.
	– Improved risk management.	– Responsible use of water.	– Price premium.	– Development of human capital.
	– Tecnologic innovation.	– Enhanced understanding of environmental issues.	– Traceability System (guarantee of origin).	– Greater job security.
	– Alignment with legislation and greater understanding of it.	– Search for cleaner energies.	– Acceptance and access to the product in new markets and cultures.	– Greater access to knowledge and technical support.
	– Changes in capabilities, practices, processes, relationships, opportunities.	– Increased monitoring and faster response to impacts.	– Responding to customer demands on a more regular basis.	– Worldwide dissemination of a product typical of the Amazon and extractive communities.
	– Alignment with internal management systems and processes.	– Focus on organic agricultural production.	– Increased partnerships with longer contracts and equitable clauses.	– Development of extractive communities.
	– Due diligence.		– Quality improvement.	
	– Active voice in political dialogues with the participation of NGOs, the public sector and academia.			
**SUPPORT MECHANISMS**				

### 3.8 SWOT analysis

The results of the case studies can also be grouped and synthesized through the SWOT Matrix (Table 5), which shows the main Strengths, Weaknesses, Opportunities, and Threats related to VSS in the global açai value chain.

**Table 5:** SWOT matrix related to VSS in the global açaí value chain.

**INTERNAL CONTEXT**	**Strengths**	**Weaknesses**
	1) They follow the legislation and regulations, complementing them.	1) Risks to the stakeholders that are in the agricultural links of the chain.
	2) They support measures to reduce informality.	2) Insufficient information on impacts of VSS.
	3) They encourage R&D, innovation and technologies.	3) Lack of clarity in incentives for local producers.
	4) Facilitate supply agreements and contracts.	4) Greenwashing.
	5) They can minimize environmental problems.	5) Açaí monoculture.
	6) Adding value.	6) Little progress in the involvement of government bodies.
	7) Improvement of production processes.	7) Enhancement of the local product.
	8) They guarantee the quality of the fruit and production.	8) High adoption and maintenance costs.
	9) Reduction of middlemen.	9) Valuing quantity and not necessarily quality.
	10) They can provide improvement in regional education.	10) Youth rural exodus.
	11) They guarantee the reliability of the processes.	11) Unfair distribution between links.
	12) They provide implementation of more sustainable practices.	12) Risk of extinction of traditional açaí mixers.
	13) They promote the development of effective methodologies.	13) Price increase.
	14) They avoid incorrect forest management.	
	15) Improve technical skills and knowledge.	
	16) Provide improvements in working conditions.	
	17) They help with public policies and economic incentives.	
	18) They bring requirements aimed at sustainability.	
	19) Increase customer satisfaction and loyalty.	
**EXTERNAL CONTEXT**	**Opportunities**	**Threats**
	1)Influences of global markets.	1) They cause geographical dispersion of the links in the chain.
	2) Improvement in the supply-demand ratio	2) Cause market barrier.
	3) Disseminate more information on the açaí GVC.	3) Risk of açaí becoming a commodity.
	4) Financing made easy.	4) Land disputes.
	5) They promote the integration of diverse stakeholders.	5) Lack of governance.
	6) They raise consumer awareness of sustainable issues.	
	7) Generate more financial resources.	
	8) New market niches.	
	9) Premium price.	
	10) Greater verticalization of GVC.	
	11) Logistic efficiency.	

## 4 Discussion

The results showed that the Voluntary Sustainability Standards include requirements on a variety of themes that the açaí enterprises need to know and fulfill to be considered sustainable.

In addition to this are changes in the structure and processes of firms, which involve investments, production costs, and the need to interact with different stakeholders. This corroborates the authors [11,29,30], which highlighted this range of heterogeneous metrics that enterprises are required to meet. This range of indicators needs to be simplified for better deployment and implementation, especially by small producers.

Generally, the representatives of the açaí companies perceive the VSS as positive for sustainability in their enterprises and mentioned successful actions in the four dimensions of sustainability. However, the survey points out that such statements and perceptions are questionable when these groups self-assess themselves on each of the indicators and themes addressed. In addition, they are susceptible to opacity and with possibilities for greenwashing practices.

Although the role of VSS is to help companies review procedures, establish sustainability goals, and implement actions in the different dimensions, it was still noticeable in the interviews that companies have difficulties in measuring their impacts and especially in disclosing the negative results. This is in line with many researchers, such as [6,7,12], and reports in the media about the fears, omissions, or practices of greenwashing.

The evaluation of the micro-enterprise, for example, reveals many positive points in the four dimensions surveyed, which the company noted when it started to adapt its processes to the requirements of the VSS it aims to adopt. Some of these results are similar to or higher than those of the other companies in the study that already have VSS. However, the micro-enterprise has still not been audited and therefore has no evidence of the conformities and nonconformities of its actions.

The fact that the micro-enterprise is in the phase of adapting to the requirements of some VSS has influenced its results. The absence of VSS in this firm, so far, is due to the financial issues of the micro-enterprise, which would need the encouragement of large industries to achieve the adoption of the certifications [18]. [31] also found in her study results of non-certified processes better than others with VSS, as well as this need for external help for adoption. This shows that in some indicators, the micro-enterprise has already implemented sustainable practices and succeeded without yet being certified. However, to achieve certification, it would need to further adapt its processes, which would currently only be possible with external help to make the necessary improvements.

For the small Cooperative, despite the partnership with multinationals to meet the requirements, obtain the VSS, and meet the demands of the external market regarding a traceable and certified açaí, the results were minimal, especially in the environmental dimension. Accordingly, having VSS under the legal umbrella of large companies has, in this case, shown few concrete sustainable results.

The medium-sized Cooperative and the medium-sized Trading Company showed good degrees of knowledge and performance on the sustainability dimensions. In Fig. 6, it was noted that this Cooperative has the second-best evaluation among the certified companies in all four dimensions, with most of the issues in the polygon areas rated as “good” or “better” and second only to the large company. The Trading Company, in its operational context (does not process açaí, only exports), perceives as fundamental the adoption of sustainable practices via VSS to be able to reach several countries, but not as the only tool. Authors such as [32,33] have already confirmed that VSS is not a unique solution and should be seen in more comprehensive contexts.

The four cases above meet the findings of the authors [13,34] when they state the need for external support (partnerships) for the certification of small and medium producers since VSS demand investments and technical capabilities that micro, small, and medium enterprises (MSMEs) usually do not have. However, this research showed that there are cases in which, even with external support, good performance is not always guaranteed, as in the mentioned case of the small Cooperative. This agrees with [35], who stated that VSS is not a sufficient condition to improve outcomes. In other words, in addition to external help for VSS, other mechanisms and actions are needed to improve the indicators analyzed.

The large company performed best, as can be seen in the comparative results in Figs. 6, Figure 7, and Fig. 8. A large industry has the most sustainable certifications in this study and perceives VSS as drivers of a sustainable market, in which companies adopt it to increase their participation in international markets, meeting the different cultures and their requirements.

There is a consensus in the literature [[36], [37], [38], [39]] that VSS facilitates this access to large markets, even though these certifications are also seen as non-tariff barriers, especially to small producers. The study confirmed this effect in the Açai GVC, in which the medium-sized commercial company and the large industry export their products to dozens of countries around the world. However, in the case of micro and small cooperatives, they are still far from directly exporting their products and need resources and partnerships for investments. This is in line with what the authors [13] have emphasized, that is, to access certified markets, small producers need to organize themselves into cooperatives or other types of small producer groups to lower transaction costs. The medium Cooperative followed this path.

The actions reported by the açaí companies in the Governance, Environmental, Economic, and Social dimensions showed successful examples. But many results generated from this data match others identified by Ref. [40] for the same chain in the economic, social, and environmental dimensions. In her research, the author pointed out situations such as VSS rarely provide tangible benefits for communities, often being only a prerequisite for entering the markets; the market has only a commercial objective for the VGC, it does not value the environmental part; the chain has improved livelihoods, but in no way reshaped the systemic biases of value distribution; there are rigid contractual sales agreements, but they become particularly vulnerable when the volatility of the raw material increases.

Such differences and variations of sustainable results in each dimension found for the açaí GVC are also found in other chains, corroborating many researchers [2]. noticed positive results in the social and environmental dimensions of the coffee chain but also negative impacts or low scores in the economic and governance dimensions [14]. evidenced positive and negative results in the coffee chain in environmental aspects, but depending on the country. For example [41], well evaluated this same dimension in the sugar cane chain.

This means that the diverse results on the dimensions of sustainability found in this research are related to the numerous methodologies employed to assess the impacts of VSS; differences in activities among chains; how GVCs are structured; their location, and the level of coverage of certified companies that the chain has, in addition to the VSS that each GVC adopts, which also vary in scope and metrics. This result confirms what the studies by Refs. [[42], [43], [44], [45]] have highlighted, the difficulty for VSS to achieve effective results across all dimensions of sustainability.

The finding that the MSMEs in this study had the lowest scores and that they were only able to join VSS and enter the GVC with the help of the large ones in the sector becomes controversial when VSS are seen as instruments for sustainable improvement, inclusion, and voluntary application. The results point out that for the açaí chain, such standards are becoming mandatory, with a low probability of companies entering international trade without sustainable certifications. It was also possible to highlight the great distance that these weaker elements of the açaí chain must travel to meet the various requirements. They are based on bureaucratic managerial activities, which are extraordinarily complex and involve many themes, sub-themes, and indicators.

Regarding large industries, other challenges refer mainly to issues related to the traceability of the fruit, investment in agro-extraction suppliers, and the search for good relationships with the extraction communities. Despite not solving the basic problems of these communities (education, health, sanitation, employment), the adoption of VSS by these large companies provides investments in projects in the communities. For places where public services do not exist or are barely offered, it is already a differential.

The agro-extractivist, represented here by the cooperatives and the micro-enterprise, are fundamental for the chain to be considered sustainable. However, despite being the main agents, they remain as supporting actors. It was found that the VSS in the açaí GVC provides opportunities for the inclusion of these agents in the global economy through partnerships, but, at the same time, they demand a lot and do not offer it in the same proportion. To ensure the sustainability of the açaí chain, the work of these actors at the base must be better valued economically.

The study identified the improvements perceived by the companies participating in the study when adopting VSS. These improvements, summarized in Table 4, show the sustainable results with the adoption of VSS for the açaí chain and are complemented by the SWOT Matrix (Table 5), which, in addition to the positive results (strengths and opportunities), also shows the weaknesses and threats of VSS for this sector chosen in the study.

Also evidenced in the mapping was an exaggerated amount of VSS that the market “requests” for açai entry into different nations. Examples of excess are related to more than one organic VSS, or in the field of food safety, or sustainable management together with chain of custody (traceability) certification.

Thus, the results suggest that VSS is a consequence of international trade requirements, not a cause of sustainability in the açaí chain. VSS is one mechanism among many others for achieving sustainability. The testimonies confirm that VSS is a result of a market imposition process, which leads to the natural improvement of the processes to become sustainable, whenever the standards are effectively met and verified.

The results also reveal that the expectation of economic gains and the reach of different markets synthesize as the great stimulus for adhesion to VSS. None of the four certified companies in the açaí GVC had limited or poor results in the economic dimension or the establishment non-certified.

In all scenarios evaluated, the environmental dimension was behind in the results of the other dimensions, as also found by Ref. [13]. In this sense, the sustainable meaning focused on the environmental dimension, which should be the priority for the maintenance of the standing forest, is not what stimulates the involvement of the companies of the açaí chain with the VSS.

The VSS generally has a strong impact on some indicators, sub-themes, or sustainable themes. But as seen in the case studies, adherence to many VSS is necessary for better company results. This explains the dynamic and unrestrained adoption of various VSS by the açai companies to satisfy different consumer audiences and countries and to demonstrate a high or at least satisfactory degree of sustainability in their products. The VSS is a guide to the search for sustainability in this GVC but not the solution. The different certifications are far from achieving the maintenance of standing forests on their own. VSS only accounts for a fraction of this purpose.

## 5 Conclusions

There is a huge challenge for VSS to provide requirements that really will bring sustainable and feasible results for all interested parties. This research shows how complex the assessment of the impacts of VSS is in global value chains such as açaí. The evaluation involved a diversification of themes, analysis, and the need for several indicators.

The first point to consider is the collective barriers of VSS. These barriers involve global value chains in general and are related to future assessment of the results provided by VSS in achieving the goals of Agenda 2030 and in the sustainability information in the impact reports released by companies if there are or not practices of *greenwashing, greenblushing*,^1^ and *greenbashing*.^2^

The integration of the data from the description, the analysis of the parties, the perceptions of the companies, and their successful actions, associated with internal and external factors to the GVC, on the perspective of the VSS provides new information to describe the impacts of the VSS on the açaí chain in the four dimensions of sustainability. Access to this information helps different-sized companies, government agencies, NGOs, and Certifiers to better understand the VSS, establish strategies for the VGC, and debate among these diverse interested parties that will be able to articulate and extract the strength points to mitigate the risks. The information can contribute to a sustainability transition with structural changes in the açaí chain.

For the different parts of the chain, it appears that the common obstacles related to the VSS issue involve compliance with numerous sustainable requirements. In addition, there is the need for constant knowledge updating at the most varied hierarchical levels and the responsibility to monitor and keep records that show compliance with the requirements of the norms.

The interdisciplinary approach to VSS points out the interdependence of key variables that drive sustainability in the açai GVC. Thus, the conclusion is that VSS-based certifications are a tool that seeks to bring together various areas of knowledge to ensure that sustainable practices are deployed and implemented in the chain. These sustainable practices are difficult to evaluate, to verify and still vary according to the size of the company and the link in which it is located.

For the açaí chain, VSS contributes to structural changes that, in the future, may provide results that make it a fully sustainable chain. However, additions such as those that ensure credibility and solve greenwashing problems are lacking. The conclusion is that the VSS are drivers for this GVC to improve its results in the four dimensions of this study and trace sustainable paths that involve all parts of the chain.

In the specific case of the açaí GVC, one can wonder about the long-term sustainability of VSS since the adoption of VSS is dynamic in this chain. Companies are constantly adding more VSS. The impacts of VSS are varied and diversified in the dimensions studied and in the different parts of the chain of this non-timber forest product. However, the continued increase in certified companies will provide a standing and sustainable Amazon forest? In this sense, among several suggestions for future studies, there is the need to monitor, over time, the application of these certifications. Most of the cases included in this research have adopted VSS in recent years.

## Data availability statement

The data are included in the article and supplementary material.

## CRediT authorship contribution statement

**Kairo Fernandes Martins:** Writing – review & editing, Writing – original draft, Visualization, Validation, Project administration, Methodology, Investigation, Formal analysis, Data curation, Conceptualization. **Denilson Teixeira:** Writing – review & editing, Validation, Supervision, Resources, Formal analysis.

## Declaration of competing interest

The authors declare that they have no known competing financial interests or personal relationships that could have appeared to influence the work reported in this paper.

## Figures and Tables

**Table:** 

Sustainability Dimension G: GOOD GOVERNANCE		
Themes	Sub-Themes	Default Indicators
G1 Corporate Ethics	G1.1 Mission Statement	G 1.1.1 Mission Explicitness
		G 1.1.2 Mission Drive
	G 1.2 Due Diligence:	G 1.2.1 Due Diligence:
G2 Accountability	G 2.1 Holistic Audits	G 2.1.1 Holistic Audits
	G 2.2 Responsibility	G 2.2.1 Responsibility:
	G 2.3 Transparency	G 2.3.1 Transparency
G3 Participation	G 3.1 Stakeholder Dialogue	G 3.1.1 Stakeholder Identification
		G 3.1.2 Stakeholder Engagement
		G 3.1.3 Engagement Barriers
		G 3.1.4 Effective Participation
	G 3.2 Grievance Procedures	G 3.2.1 Grievance Procedures
	G 3.3 Conflict Resolution	G 3.3.1 Conflict Resolution
G4 Rule of Law	G 4.1 Legitimacy	G 4.1.1 Legitimacy
	G 4.2 Remedy, Restoration and Prevention	G 4.2.1 Remedy, Restoration and Prevention
	G 4.3 Civic Responsibility	G. 4.3.1 Civic Responsibility
	G 4.4 Resource Appropriation	G 4.4.1 Free, Prior and Informed Consent
		G 4.4.2 Tenure Rights
G5 Holistic Management	G 5.1 Sustainability Management Plan	G 5.1.1 Sustainability Management Plan
	G 5.2 Full-Cost Accounting	G 5.2.1 Full-Cost Accounting

**Table:** 

Sustainability Dimension E: ENVIRONMENTAL INTEGRITY		
Themes	Sub-Themes	Default Indicators
E1 Atmosphere	E 1.1 Greenhouse Gases	E 1.1.1 GHG Reduction Target
		E 1.1.2 GHG Mitigation Practices
		E 1.1.3 GHG Balance
	E 1.2 Air Quality	E 1.2.1 Air Pollution Reduction Target
		E 1.2.2 Air Pollution Prevention Practices
		E 1.2.3 Ambient Condentration of Air Pollutants
E2 Water	E 2.1 Water Withdrawal	E 2.1.1 Water Conservation Target
		E 2.1.2 Water Conservation Practices
		E 2.1.3 Ground and Surface Water Withdrawals
	E. 2.2 Water Quality	E 2.2.1 Clean Water Target
		E 2.2.2 Water Pollution Prevention Practice
		E 2.2.3 Concentration of Water Pollutants
		E 2.2.4 Wastewater quality
E3 Land	E 3.1 Soil Quality	E 3.1.1 Soil Improvement practices
		E 3.1.2 Soil Physical Structure
		E 3.1.3 Soil Chemical Quality
		E 3.1.4 Soil Biological Quality
		E 3.1.5 Soil Organic Matter
	E 3.2 Land Degradation	E 3.2.1 Land Conservation and Rehabilitation Plan
		E 3.2.2 Land Conservation and Rehabilitation Practices
		E 3.2.3 Net Loss/Gain of Productive Land
E4 Biodiversity	E 4.1 Ecosystem Diversity	E 4.1.1 Landscape/Marine Habitat Conservation Plan
		E 4.1.2 Ecosystem Enhancing Practices
		E 4.1.3 Structural Diversity of Ecosystems
		E 4.1.4 Ecosystem Connectivity
		E 4.1.5 Land Use and Land Cove Change
	E 4.2 Species Diversity	E 4.2.1 Species Conservation Target
		E 4.2.2 Species Conservation practices
		E 4.2.3 Diversity and Abundance of Key Species
		E 4.2.4 Diversity of Production
	E. 4.3 Genetic Diversity	E 4.3.1 Wild Genetic Diversity Enhancing Practices
		E 4.3.2 Agro-biodiversity in-situ Conservation
		E 4.3.3 Locally Adapted Varieties/Breeds
		E 4.3.4 Genetic Diversity in Wild Species
		E 4.3.5 Saving of Seeds and Breeds
E5 Materials and Energy	E 5.1 Material Use	E 5.1.1 Material Consumption Practices
		E 5.1.2 Nutrient Balances
		E 5.1.3 Renewable and Recycled Materials
		E 5.1.4 Intensity of Material Use
	E 5.2 Energy Use	E 5.2.1 Renewable Energy Use Target
		E 5.2.2 Energy Saving Practices
		E 5.2.3 Energy Consumption
		E 5.2.4 Renewable Energy
	E 5.3 Waste Reduction and Disposal	E 5.3.1 Waste Reduction Target
		E 5.3.2 Waste Reduction Practices
		E 5.3.3 Waste Disposal
		E 5.3.4 Food Loss and Waste Reduction
E6 Animal Welfare	E 6.1 Animal Health	E 6.1.1 Animal Health Practices
		E 6.1.2 Animal Health
	E 6.2 Freedom from Stress	E 6.2.1 Humane Animal Handling Practices
		E 6.2.2 Appropriate Animal Husbandry
		E 6.2.3 Freedom from Stress

**Table:** 

Sustainability Dimension C: ECONOMIC RESILIENCE		
Themes	Sub-Themes	Default Indicators
C1 Investment	C 1.1 Internal Investment	C 1.1.1 Internal Investment
	C 1.2 Community Investment	C 1.2.1 Community Investment
	C 1.3 Long Ranging Investment	C 1.3.1 Long Term Profitability
		C 1.3.2 Business Plan
	C 1.4 Profitability	C 1.4.1 Net Income
		C 1.4.2 Cost of Production
		C 1.4.3 Price Determination
C2 Vulnerability	C 2.1 Stability of Production	C 2.1.1 Guarantee od Production Levels
		C 2.1.2 Product Diversification
	C 2.2 Stability of Supply	C 2.2.1 Procurement Channels
		C 2.2.2 Stability of Supplier Relationship
		C 2.2.3 Dependence on the Leading Supplier
	C 2.3 Stability of Market	C 2.3.1 Stability of Market
	C 2.4 Liquidity	C 2.4.1 Net Cash Flow
		C 2.4.2 Safety Nets
	C 2.5 Risk Management	C 2.5.1 Risk Management
C3 Product Quality and Information	C 3.1 Food Safety	C 3.1.1 Control Measures
		C 3.1.2 Hazardous Pesticides
		C 3.1.3 Food Contamination
	C 3.2 Food Quality	C 3.2.1 Food Quality
	C 3.3 Product Information	C 3.3.1 Product Labelling
		C 3.3.2 Traceability Systems
		C 3.3.3 Certified Production
C4 Local Economy	C 4.1 Value Creation	C 4.1.1 Regional Workforce
		C 4.1.2 Fiscal Commitment
	C 4.2 Local Procurement	C 4.2.1 Local Procurement

**Table:** 

Sustainability Dimension S: SOCIAL WELL-BEING		
Themes	Sub-Themes	Default Indicators
S1 Decent Livelihood	S 1.1 Quality of Life	S 1.1.1 Right to Quality of Life
		S 1.1.2 Wage Level
	S 1.2 Capacity Development	S 1.2.1 Capacity Development
	S 1.3 Fair Access to Means of Production	S 1.3.1 Fair Access to Means of Production
S2 Fair Trading Practices	S 2.1 Responsible Buyers	S 2.1.1 Fair Pricing and Transparent Contracts
	S 2.2 Rights of Suppliers	S 2.2.1 Rights of Suppliers
S3 Labour Rights	S 3.1 Employment Relations	S 3.1.1 Employment Relations
	S 3.2 Forced Labour	S 3.2.1 Forced Labour
	S 3.3 Child Labour	S 3.3.1 Child Labour
	S 3.4 Freedom of Association and Right to Bargaining	S 3.4.1 Freedom of Association and Right to Bargaining
S4 Equity	S 4.1 Non Discrimination	S 4.1.1 Non Discrimination
	S 4.2 Gender Equality	S 4.2.1 Gender Equality
	S 4.3 Support to Vulnerable People	S 4.3.1 Support to Vulnerable People
S5 Human Safety and Health	S 5.1 Workplace Safety and Health Provisions	S 5.1.1 Safety and Health Training
		S 5.1.2 Safety of Workplace, Operations and Facilities
		S 5.1.3 Health Coverage and Access to Medical Care
	S 5.2 Public Health	S 5.2.1 Public Health
S6 Cultural Diversity	S 6.1 Indigenous Knowledge	S 6.1.1 Indigenous Knowledge
	S 6.2 Food Sovereignty	S 6.2.1 Food Sovereignty

## Footnotes

1 Term used for companies that are silent to understate or ignore their commitment and actions on corporate social and environmental responsibility, telling the minimum or only about what is questioned, avoiding the most difficult or controversial themes [46].

2 Term to express products that have good environmental performance but are poorly evaluated [33].
